# The impact of prior malignancies on the development of second malignancies and survival in follicular lymphoma: A population‐based study

**DOI:** 10.1002/jha2.108

**Published:** 2020-10-08

**Authors:** Manette A.W. Dinnessen, Otto Visser, Sanne H. Tonino, Marjolein W.M. van der Poel, Nicole M.A. Blijlevens, Marie José Kersten, Pieternella J. Lugtenburg, Avinash G. Dinmohamed

**Affiliations:** ^1^ Department of Research and Development Netherlands Comprehensive Cancer Organisation (IKNL) Utrecht The Netherlands; ^2^ Department of Registration Netherlands Comprehensive Cancer Organisation (IKNL) Utrecht The Netherlands; ^3^ Department of Hematology Cancer Center Amsterdam LYMMCARE (Lymphoma and Myeloma Center Amsterdam) Amsterdam UMC University of Amsterdam Amsterdam The Netherlands; ^4^ Department of Internal Medical Division of Hematology Maastricht University Medical Center Maastricht The Netherlands; ^5^ Department of Hematology Radboud University Medical Center Nijmegen The Netherlands; ^6^ Department of Hematology Erasmus MC Cancer Institute Rotterdam The Netherlands; ^7^ Amsterdam UMC Vrije Universiteit Amsterdam Department of Hematology Cancer Center Amsterdam Amsterdam The Netherlands; ^8^ Department of Public Health Erasmus University Medical Center Rotterdam The Netherlands

**Keywords:** follicular lymphoma, non hodgkin lymphoma, second malignancy, risk factor, treatment‐related neoplasm, epidemiology, registry

## Abstract

We assessed the impact of a prior malignancy diagnosis (PMD) – as a potential proxy for genetic cancer susceptibility – on the development of a second primary malignancy (SPM) and mortality in follicular lymphoma (FL) patients. From the nationwide Netherlands Cancer Registry, we selected all adult FL patients diagnosed in 1994‐2012 (n = 8028) and PMDs and SPMs relative to FL, with follow‐up until 2017. We constructed two Fine and Gray models – with death as a competing risk – to assess the association between a PMD and SPM incidence. A PMD was associated with an increased incidence of SPMs (subdistribution hazard ratio [SHR], 1.30; 95% confidence interval [CI], 1.03‐1.64) – especially carcinomas of the respiratory tract (SHR, 1.83; 95% CI, 1.10‐3.05) and cutaneous squamous cell carcinomas (SHR, 1.58; 95% CI, 1.01‐2.45) – and a higher risk of mortality in a multivariable model (HR, 1.43; 95% CI, 1.19‐1.71). However, when additionally adjusted for the receipt of systemic therapy and/or radiotherapy before FL diagnosis, only patients who received such therapies had an increased incidence of SPMs (SHR, 1.40; 95% CI, 1.02‐1.93). In conclusion, patients with a PMD had a higher rate of SPMs and mortality than those without a PMD, which might have resulted from therapy‐related carcinogenesis.

## 1 INTRODUCTION

Advances in the diagnosis and management of follicular lymphoma (FL) – most notably the introduction of rituximab – have considerably improved the survival of patients with FL over the past decades [1, 2, 3, 4, 5, 6, 7, 8, 9]. In the present chemoimmunotherapy era, 5‐year relative survival rates for newly diagnosed patients with FL range between 41% and 95%, depending on the sex, race, age, disease stage, and geographical location [5, 6, 8, 9, 10, 11].

The improved longevity of patients with FL might come at a price, as these patients might live long enough to develop second primary malignancies (SPMs). A few studies have reported an increased risk of hematological and solid SPMs among patients with FL, as compared to the general population [12, 13, 14]. More specifically, patients had a statistically significantly elevated risk of Hodgkin lymphoma and acute myeloid leukemia, and solid tumors of the following sites: oral cavity and pharynx, stomach, lung, melanoma skin, nonmelanoma skin, urinary bladder, and kidney/pelvis [12, 13, 14].

SPMs could reflect late sequelae of treatment or the effect of shared etiologic factors, environmental exposures, and genetic and non‐genetic host characteristics, as well as combinations of these influences – including gene‐environment and gene‐gene interactions. Suggested risk factors for SPMs among patients with FL [12, 13, 14] and non‐Hodgkin lymphoma in general, included age > 65 years, male sex, and receipt of radio‐ and/or chemotherapy for the lymphoma [15].

Among patients with multiple myeloma diagnosed in Sweden, a prior malignancy diagnosis (PMD) – as a potential proxy for genetic susceptibility to cancer – was associated with SPM development and mortality, as compared to those without a PMD [16]. At present, information on the impact of a PMD on the development of SPMs and mortality among patients with FL is lacking. This information is important as it could have consequences for surveillance on the development of SPMs among patients with a PMD. Also, whenever a newly diagnosed patient with FL has received prior anti‐neoplastic therapy, a treatment regimen with a low toxicity profile might be considered to avoid excessive, cumulative toxicity and reduce treatment‐related mortality. Therefore, this nationwide population‐based study aimed to assess the impact of a PMD on the development of SPMs and mortality in patients with FL in the Netherlands.

## 2 METHODS

### 2.1 The Netherlands Cancer Registry

Established in 1989, the nationwide population‐based Netherlands Cancer Registry (NCR), which is managed by the Netherlands Comprehensive Cancer Organisation (IKNL), covers at least 95% of all newly diagnosed malignancies in the Netherlands [17]. The NCR is notified by the Nationwide Network and Registry of Histopathology and Cytopathology, and the National Registry of Hospital Discharges (ie, inpatient and outpatient discharges). Information on dates of birth and diagnosis, sex, disease topography and morphology, and broad categories of primary therapy started within the first 9‐12 months after diagnosis is routinely recorded in the NCR by trained registrars of the NCR through retrospective medical records review. Topography and morphology are coded according to the International Classification of Diseases for Oncology. Data on vital statistics (ie, alive, death, or emigration) are retrieved through annual linkage with the Nationwide Population Registries Network that holds these data for all residents in the Netherlands.

### 2.2 Study population

**FIGURE 1:**
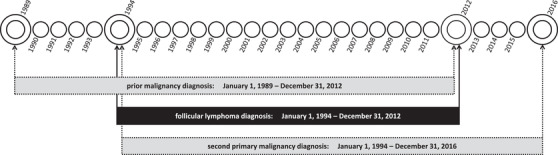
Patient selection and selection of prior and subsequent malignancies

We selected all adult (≥18 years) patients diagnosed with FL grades 1–3B between January 1, 1994 and December 31, 2012, from the NCR. FL was defined as per the third edition of the International Classification of Diseases for Oncology morphology codes 9693 and 9697 for patients diagnosed from 1989 to 2001 and 9690, 9691, 9695, and 9698 for patients diagnosed from 1989 onward [18]. PMDs that were diagnosed between January 1, 1989 and December 31, 2012, and SPMs that were diagnosed between January 1, 1994 and December 31, 2016, were identified by cross‐linkage with the NCR. This selection strategy allowed for at least 5 years of follow‐up to capture a PMD and to develop an SPM. The selection of PMDs and SPMs relative to FL is depicted in Figure 1. Benign, borderline, in situ, and basal cell carcinomas were excluded. PMDs and SPMs were classified into the following subtypes as per the third edition of the International Classification of Diseases for Oncology: (a) bone and soft tissue; (b) breast; (c) endocrine; (d) female reproductive; (e) gastrointestinal; (f) head and neck; (g) hematological; (h) kidney and urinary tract; (i) male reproductive; (j) melanoma of the skin; (k) nervous system; (l) respiratory tract; (m) squamous cell of the skin; and (n) unspecified. Patients diagnosed with FL at autopsy (n = 19) and patients with synchronous malignancies within a time‐interval of 3 months before or after FL diagnosis (n = 467) were excluded. We excluded patients with synchronous malignancies, as malignancies diagnosed simultaneously to FL might be detected coincidentally, thereby not truly reflecting a PMD or SPM.

According to the Central Committee on Research involving Human Subjects (CCMO), this type of observational study does not require approval from an ethics committee in the Netherlands. The use of anonymous data for this study was approved by the Privacy Review Board of the NCR.

### 2.3 Statistical analysis

Descriptive statistics were employed to compare patient characteristics between those with and without a PMD. The Pearson chi‐square test was applied to compare categorical variables. The Mann‐Whitney *U* test was used to compare continuous variables.

We constructed two competing risk regression models as per Fine and Gray to estimate subdistribution hazard ratios (SHRs) with 95% confidence intervals (CIs) for the association between a PMD and the diagnosis of a first SPM. In Model 1, the exposure was the binary variable of a PMD before FL diagnosis (no *versus* yes). In Model 2, patients with a PMD were classified as patients (a) with or (b) without receipt of systemic therapy and/or radiotherapy before the diagnosis of FL. Consequently, effect estimates for the latter category might provide clues on the effect of other factors associated with SPM development, such as genetic susceptibility or sequelae of prior cancer therapy. Death before the diagnosis of an SPM was regarded as a competing risk. In the absence of an event, patients were censored at the time of emigration or at the end of the study (ie, December 31, 2016), whichever occurred first. Both models were additionally adjusted for potential confounders, namely sex, age at FL diagnosis, year of FL diagnosis, and stage at FL diagnosis. SHRs for the association between a PMD and the diagnosis of a first SPM were also calculated for subtypes of SPMs by using Model 1.

The SHR describes the relative change in the instantaneous *rate* of the occurrence of an SPM in those who did not develop an SPM during follow‐up (ie, the event of interest) and those who died before that event occurred (ie, the competing risk) [19]. Given the relationship with the cumulative incidence function for the subdistribution hazard function, the SHRs can also be interpreted as the effect of a PMD on the *incidence* of SPMs. Of note, the magnitude of the effect of a PMD on the incidence of SPMs cannot be directly quantified by using SHRs.

Similar to the competing risk models, we constructed two Cox proportional hazard models to calculate hazard ratios (HRs) with 95% CIs for the association between a PMD and mortality (ie, overall survival). Patients were censored at the time of emigration or at the end of the study (ie, December 31, 2016), whichever occurred first. Both models were additionally adjusted for baseline characteristics at FL diagnosis, namely sex, age at diagnosis, year of diagnosis, and stage at diagnosis.

We performed sensitivity analyses in which we excluded patients with synchronous malignancies within a time‐interval of 6 months before or after FL diagnosis. The impact of applying different definitions of synchronous malignancies in relation to the outcome has been appraised previously [20, 21].

A *P*‐value of <.05 indicated statistical significance. Statistical analyses were performed with STATA Statistical Software version 14.2 (StataCorp, College Station, TX).

## 3 RESULTS

### 3.1 Patient characteristics

**FIGURE 2:**
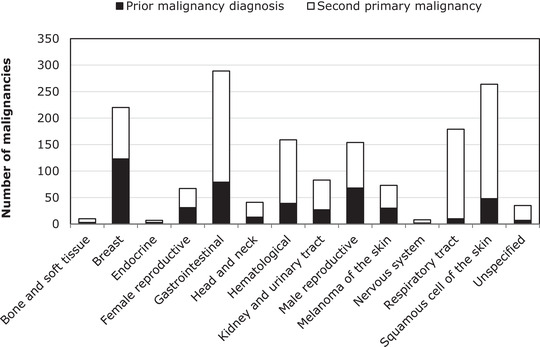
Types of prior and subsequent malignancies among patients with follicular lymphoma. The absolute number of prior and subsequent malignancies according to type is also presented in Supplemental Table 3

A total of 8028 patients with FL—of whom 483 (6%) had a PMD and 1106 (14%) developed an SPM—were included in the study. Characteristics of these patients at the time of FL diagnosis are presented in Table 1 according to the history of a PMD. The majority of patients with a PMD did not receive systemic therapy and/or radiotherapy for the treatment of their PMD (52%; Supplemental Table 1). Lastly, patients with a PMD were more often female (57% *versus* 50%; *P <* 0.001) and older at FL diagnosis (median age 69 *versus* 60 years; *P = *0.005), as compared to patients without a PMD. The specific subtypes of PMDs and SPMs are presented in Figure 2.

**TABLE 1:** Patient characteristics

	FL with a PMD		FL without a PMD		Total	
Patients, n (% row)	483	(6.0)	7545	(94.0)	8028	(100)
Patients with an SPM, n (%)	83	(17.2)	1023	(13.6)	1106	(13.8)
Median age at FL diagnosis, years (IQR)	69.3	(61.3‐76.8)	60.1	(51.0‐69.3)	60.7	(51.4‐69.9)
Male sex, n (%)	209	(43.3)	3765	(49.9)	3,974	(49.5)
Calendar period of FL diagnosis, n (% row)						
1994‐1998	38	(2.4)	1529	(97.6)	1567	(100)
1999‐2002	73	(4.6)	1502	(95.4)	1575	(100)
2003‐2008	187	(6.6)	2635	(93.4)	2822	(100)
2009‐2012	185	(9.0)	1879	(91.0)	2064	(100)
Median time from first PMD to FL, years (IQR)						
Total	5.3	(2.2‐9.3)	–	–	–	–
With an SPM	5.3	(2.7‐9.3)	–	–	–	–
Without an SPM	5.4	(2.1‐9.3)	–	–	–	–
Median follow‐up time after FL diagnosis, years (IQR)	5.5	(3.0‐8.4)	7.2	(4.2‐11.5)	7.1	(4.2‐11.3)

Abbreviations: FL, follicular lymphoma; IQR, interquartile range; PMD, prior malignancy diagnosis; SPM, second primary malignancy.

### 3.2 Association between a PMD and SPM development

**FIGURE 3:**
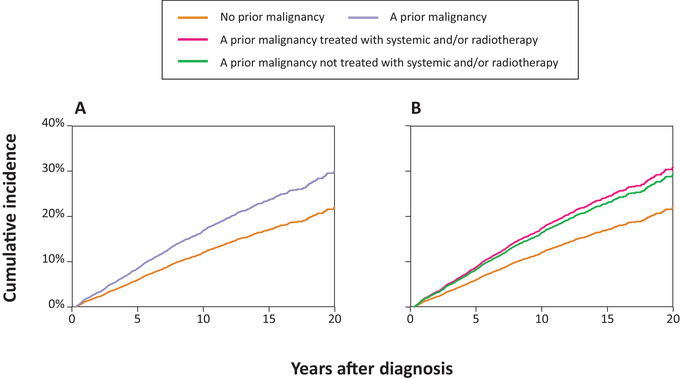
Cumulative incidence function of second primary malignancies after follicular lymphoma. In panel (A), the exposure was the binary variable of a PMD before FL diagnosis (no versus yes). In panel (B), patients with a PMD were classified as patients (i) with or (ii) without receipt of systemic or radiotherapy before FL diagnosis

The 5‐year cumulative incidence of SPMs was 5.8% (95% CI, 5.3%‐6.4%) and 10.1% (95% CI, 7.6%‐13.2%) for patients without and with a PMD, respectively (Figure 3A). In the univariable Fine and Gray regression model with PMD regarded as a binary variable, a PMD was associated with an increased incidence of SPMs (SHR, 1.44; 95% CI, 1.15‐1.80; *P *= 0.001; Table 2). Subgroup analyses revealed a higher incidence for carcinomas of the respiratory tract (SHR, 1.91; 95% CI, 1.16‐3.15; *P *= 0.011) and squamous cell carcinomas (SHR, 2.02; 95% CI, 1.31‐3.12; *P *= 0.001; Supplemental Table 2). Adjustment for baseline characteristics did not largely affect the SHRs for the overall (Model 1; Table 2) and subgroup analyses (Supplemental Table 2). Furthermore, multivariable analyses showed that male sex and age at FL diagnosis per ten‐year increase were independently associated with a greater cumulative incidence of SPMs (Model 1; Table 2). SPM incidence was not influenced by the year of FL diagnosis per one‐year increase and the disease stage of FL (Model 1; Table 2).

**TABLE 2:** Competing risk regression models for the association between a history of malignancies and the development of second primary malignancies after follicular lymphoma diagnosis in the Netherlands

	Univariable			Multivariable model 1			Multivariable model 2		
	SHR	95% CI	*P*‐value*	SHR	95% CI	*P*‐value*	SHR	95% CI	*P*‐value*
**Prior malignancy diagnosis**									
No	1	Reference		1	Reference		–	–	–
Yes	1.44	1.15‐1.80	**.001**	1.30	1.03‐1.64	**.027**	–	–	–
**Prior malignancy diagnosis**									
No	1	Reference		–	–	–	1	Reference	
Yes with ST and/or RT	1.49	1.09‐2.04	**.013**	–	–	–	1.40	1.02‐1.93	**.039**
Yes without ST and/or RT	1.40	1.02‐1.90	**.035**	–	–	–	1.21	0.88‐1.67	.233
**Sex**									
Female	1	Reference		1	Reference		1	Reference	
Male	1.24	1.11‐1.40	**<.001**	1.32	1.17‐1.49	**<.001**	1.32	1.17‐1.48	**<.001**
**Age at FL diagnosis, 10 years**	1.16	1.12‐1.20	**<.001**	1.17	1.12‐1.22	**<.001**	1.17	1.12‐1.22	**<.001**
**Year of FL diagnosis**	1.00	0.99‐1.01	.558	1.00	0.99‐1.01	.529	1.00	0.98‐1.01	.524
**Stage of FL at diagnosis**									
I	1	Reference		1	Reference		1	Reference	
II	1.08	0.89‐1.30	.452	1.07	0.88‐1.29	.513	1.07	0.88‐1.29	.519
III	0.88	0.73‐1.04	.136	0.90	0.75‐1.07	.243	0.90	0.75‐1.07	.240
IV	0.96	0.82‐1.12	.610	1.00	0.85‐1.16	.957	1.00	0.85‐1.16	.961
Unknown	0.75	0.47‐1.19	.219	0.69	0.43‐1.11	.128	0.69	0.43‐1.11	.127

* *P*‐values are compared with the reference category. Statistically significant *P*‐values (*P *< .05) are presented in bold.

Abbreviations: CI, confidence interval; FL, follicular lymphoma; RT, radiotherapy.; SHR, subdistribution hazard ratio; ST, systemic therapy.

We specifically assessed the contribution of systemic therapy and/or radiotherapy for a PMD on SPM development. The 5‐year cumulative incidence of SPMs after FL diagnosis was 10.4% (95% CI, 6.8%‐15.1%) and 9.9% (95% CI, 6.5%‐14.3%) for patients with a PMD who were previously treated and not treated with systemic therapy and/or radiotherapy, respectively (Figure 3B). The univariable Fine and Gray regression model demonstrated that the association of a PMD with an increased incidence of SPMs was irrespective of whether a PMD was treated with systemic therapy and/or radiotherapy (Table 2). However, when adjusted for potential confounding factors, only patients with a PMD who were previously treated with systemic therapy and/or radiotherapy—as compared to those without a PMD—had a statistically significant increased incidence of SPMs (SHR, 1.40; 95% CI, 1.02‐1.93; *P *= 0.039; Model 2; Table 2). Besides, the remaining covariates associated with the cumulative incidence of SPMs in Model 2 were comparable to those observed in Model 1 (Table 2). Sensitivity analyses showed results that were comparable to the results of the primary analyses (data not shown).

### 3.3 Association between a PMD and mortality

**FIGURE 4:**
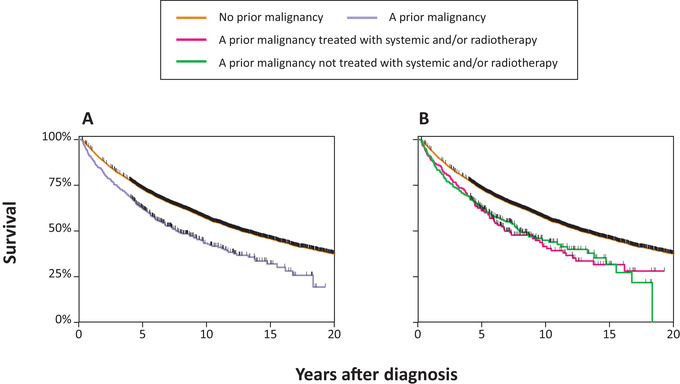
Overall survival of patients with follicular lymphoma. Panel (A) presents Kaplan‐Meier estimates of overall survival in which the exposure was the binary variable of a PMD before FL diagnosis (no vs yes). Panel (B) presents Kaplan‐Meier estimates of overall survival in which patients with a PMD were classified as patients (i) with or (ii) without receipt of systemic or radiotherapy before FL diagnosis

At a median follow‐up of 7.1 years (range, 0.3‐23.0 years), 2735 (34%) patients with FL died. Five‐year overall survival was 73% (95% CI, 72%‐74%) and 62% (95% CI, 58%‐66%) for patients without and with a PMD, respectively (*P *< 0.001; Figure 4A). In the univariable Cox model with PMD regarded as a binary variable, the risk of mortality was higher in patients with a PMD, as compared to patients without a PMD (HR, 1.51; 95% CI, 1.33‐1.71; *P <* 0.001; Table 3). This association was also observed after adjustment for baseline characteristics (HR, 1.26; 95% CI, 1.11‐1.43; *P *< 0.001; Model 1, Table 3). Male sex, age per ten‐year increase, and higher disease stage at diagnosis were independently associated with a higher risk of mortality, whereas the year of FL diagnosis per one‐year increase was associated with a lower risk of mortality.

**TABLE 3:** Cox regression models for the association between a history of malignancies and mortality among follicular lymphoma patients in the Netherlands

	Univariable			Multivariable model 1			Multivariable model 2		
	HR	95% CI	*P*‐value*	HR	95% CI	*P*‐value*	HR	95% CI	*P*‐value*
**Prior malignancy diagnosis**									
No	1	Reference		1	Reference		–	–	–
Yes	1.51	1.33‐1.71	**<.001**	1.26	1.11‐1.43	**<.001**	–	–	–
**Prior malignancy diagnosis**									
No	1	Reference		–	–	–	1	Reference	
Yes with ST and/or RT	1.54	1.29‐1.84	**<.001**	–	–	–	1.43	1.19‐1.71	**<.001**
Yes without ST and/or RT	1.48	1.25‐1.76	**<.001**	–	–	–	1.13	0.95‐1.34	.175
**Sex**									
Female	1	Reference		1	Reference		1	Reference	
Male	1.00	0.94‐1.07	.891	1.26	1.18‐1.34	**<.001**	1.26	1.18‐1.34	**<.001**
**Age at FL diagnosis, 10 years**	1.74	1.69‐1.79	**<.001**	1.85	1.80‐1.91	**<.001**	1.85	1.80‐1.91	**<.001**
**Year of FL diagnosis**	0.95	0.94‐0.95	**<.001**	0.93	0.92‐0.93	**<.001**	0.93	0.92‐0.93	**<.001**
**Stage of FL at diagnosis**									
I	1	Reference		1	Reference		1	Reference	
II	1.40	1.24‐1.57	**<.001**	1.47	1.31‐1.66	**<.001**	1.47	1.31‐1.66	**<.001**
III	1.55	1.40‐1.72	**<.001**	2.04	1.83‐2.26	**<.001**	2.04	1.83‐2.26	**<.001**
IV	1.89	1.72‐2.08	**<.001**	2.41	2.19‐2.64	**<.001**	2.41	2.19‐2.65	**<.001**
Unknown	2.26	1.83‐2.78	**<.001**	1.80	1.46‐2.22	**<.001**	1.79	1.45‐2.21	**<.001**

* *P*‐values are compared with the reference category. Statistically significant *P*‐values (*P *< .05) are presented in bold.

Abbreviations: CI, confidence interval; FL, follicular lymphoma; HR, hazard ratio; RT, radiotherapy; ST, systemic therapy.

Five‐year overall survival was 61% (95% CI, 54%‐67%) and 63% (95% CI, 57%‐69%) for patients with a PMD who were previously treated and not treated with systemic therapy and/or radiotherapy, respectively (*P *= 0.001; Figure 4B). In the univariable Cox model where patients with a PMD were broken down according to the receipt of systemic therapy and/or radiotherapy, the risk of mortality was higher for patients with a PMD—as compared to those without a PMD—irrespective of whether a PMD was treated with systemic therapy and/or radiotherapy (Table 3). However, when adjusted for potential confounding factors, the association with higher mortality was only statistically significant for patients with a PMD who were treated with systemic therapy and/or radiotherapy (HR, 1.43; 95% CI, 1.19‐1.71; *P *< 0.001; Model 2; Table 3). Additional factors that were associated with mortality in Model 2 were comparable to those observed in Model 1 (Table 3). Sensitivity analyses again showed results that were comparable to the results of the primary analyses (data not shown).

## 4 DISCUSSION

In this nationwide, population‐based study, we demonstrated that FL patients with a PMD had an increased incidence of SPMs—in particular of carcinomas of the respiratory tract and cutaneous squamous cell carcinomas—as compared to patients without a PMD. Also, patients with a PMD had a higher adjusted risk of mortality, as compared to patients without a PMD. The increased incidence of SPMs and the higher risk of mortality likely resulted, in part, from therapy‐related carcinogenesis. To our knowledge, our study is the first to assess the association of a PMD with SPM development and mortality in FL.

A significantly increased incidence of lung [12, 13, 22, 23, 24, 25, 26, 27] and (nonmelanoma) skin cancer [12, 13, 22, 23, 24, 28, 29] among patients with FL and non‐Hodgkin lymphoma in general, as compared to the general population, has been noted in previous studies. Underlying mechanisms for this increase are as yet not fully understood and are likely multifactorial. Nevertheless, the authors brought forward several arguments to discuss potential etiologies. First, a suggested mechanism is the long‐term immune dysfunction related to the lymphoma and its treatment [13, 15, 25, 26, 30], as lung [31, 32, 33, 34] and skin cancer [28, 35, 36] occur more abundantly in immunosuppressed individuals. Second, an increased incidence of skin and lung cancer is suggested to be related to exposure to radiotherapy, in particular in combination with systemic therapy [12, 13]. This phenomenon is analogous to what has been observed among patients with Hodgkin lymphoma treated with radiotherapy [37, 38, 39].

To build upon the potential etiologies discussed earlier, immunosuppression and the late effects of systemic therapy and/or radiotherapy might also explain the excess risk of lung and skin cancer among FL patients with a PMD, as compared to those without a PMD. First, patients with a PMD might have prolonged immune dysfunction related to a PMD and its treatments. Second, the carcinogenic effect of systemic therapy and/or radiotherapy is dose‐dependent [26, 38, 40, 41]. Thus, whenever a PMD was treated with systemic therapy and/or radiotherapy, the cumulative dose of potential carcinogens was likely higher for patients with a PMD, as compared to those without a PMD, due to prior cancer therapy. Taken collectively, these explanations are further supported by our findings, as we only observed an elevated incidence of SPMs for patients with a PMD who were treated with systemic therapy and/or radiotherapy, as compared to patients without a PMD.

We observed that a higher risk of mortality was only present among patients with a PMD who were treated with systemic therapy and/or radiotherapy, as compared to patients without a PMD. This finding might be explained by the (late) effects from the prior systemic therapy and/or radiotherapy, potentially leading to organ dysfunction, cardiovascular disease, and death [42, 43, 44, 45, 46]. Furthermore, morbidity among FL patients with a PMD due to prior cancer therapy might hamper the optimal management of FL with radiotherapy or chemoimmunotherapy. As a result, this might increase FL‐related mortality.

The disease stage of FL at diagnosis was an independent predictor of mortality. However, this does not exclude the possibility that FL arising after a PMD might be genetically different and more aggressive compared to FL arising as a first primary malignancy. Lastly, patients with FL who developed an SPM and received systemic therapy and/or radiotherapy for a PMD might have a higher risk of mortality due to the aggressive nature of the SPM. For instance, previous studies showed that skin cancers secondary to non‐Hodgkin lymphoma are more aggressive and associated with higher skin cancer recurrence rates and increased regional metastasis and death due to skin cancer metastases [28, 36, 47].

The main strength of our study is the use of comprehensive data that are available for individual patients from a long‐running and well‐established nationwide population‐based cancer registry. Another strength is the clear distinction between a new primary malignancy and a transformation. For example, a diffuse large B‐cell lymphoma diagnosis following FL is registered in the NCR as a transformation rather than an SPM, unless it is stated in medical records that the diffuse large B‐cell lymphoma is an SPM. Further, we performed sensitivity analyses to confirm that our results were not dependent on the chosen definition for synchronous malignancies [20, 21].

Limitations mainly pertain to the lack of detailed data on prognostic factors (eg, FL International Prognostic Index), smoking, and therapy of a PMD and FL beyond 1 year after diagnosis. Therefore, residual confounding could not be ruled out. Adjustment for the primary therapy of FL was not performed, as treatment strategies are likely to have changed during the disease course depending on the clinical behavior [48], especially for patients who were initially put on a watch‐and‐wait approach (approximately 31%; data not shown). Also, the subdivision of grade 3 into grade 3A and 3B was not recorded in the NCR. Another limitation was the comparatively low number of patients with a PMD and an SPM in our study population to perform analyses within Model 2 for subtypes of SPMs and other subgroup analyses. Lastly, patients without a PMD before 1989 may have been misclassified as patients without a PMD due to left truncation, since we only had a 5‐year lead time.

In summary, in this nationwide, population‐based study, FL patients with a PMD had an increased incidence of SPMs – particularly carcinomas of the respiratory tract and cutaneous squamous cell carcinomas – and a higher risk of mortality, as compared to patients without a PMD. The mechanism behind this was likely multifactorial, albeit our data suggest that it may have resulted, in part, from therapy‐related carcinogenesis. As the longevity of patients with FL is expected to increase, physicians should be aware of SPMs within this patient population, especially among patients with a PMD who were treated with systemic therapy and/or radiotherapy. We encourage forthcoming studies to validate our study findings through analysis of population‐based cancer registry data.

## CONFLICT OF INTEREST

MJK has received research and travel support, as well as honoraria for presentations from Roche.

### AUTHOR CONTRIBUTIONS

AGD designed the study; MAWD analysed the data; OV collected the data; MAWD wrote the manuscript with contributions from all authors, who also interpreted the data, and read, commented on, and approved the final version of the manuscript.

## Supporting information

Supporting information

## Data Availability

The data that support the findings of this study are available via The Netherlands Comprehensive Cancer Organisation. These data are not publicly available and restrictions apply to the availability of the data used for the current study. However, these data are available from the authors upon reasonable request and with permission of The Netherlands Comprehensive Cancer Organisation.
