# Supplementary material for: The impact of prior malignancies on the development of second malignancies and survival in follicular lymphoma: A population‐based study
Source: EJHaem. 2020 Oct 8;1(2):489–97. doi: 10.1002/jha2.108 (PMC9175939; doi:10.1002/jha2.108)
Supplement: Supplementary file 1 — Supporting information [file JHA2-1-489-s001.docx]

**ONLINE APPENDIX**

**Title**

The impact of prior malignancies on the development of second malignancies and survival in follicular lymphoma: a population-based study

**Authors and affiliations**

Manette A.W. Dinnessen,^1^ Otto Visser,^2^ Sanne H. Tonino,^3^ Marjolein W.M. van der Poel,^4^ Nicole M.A. Blijlevens,^5^ Marie José Kersten,^3^ Pieternella J. Lugtenburg,^6^ Avinash G. Dinmohamed^1,3,7,8^

**^1^**Department of Research and Development, Netherlands Comprehensive Cancer Organisation (IKNL), Utrecht, The Netherlands; ^2^Department of Registration, Netherlands Comprehensive Cancer Organisation (IKNL), Utrecht, The Netherlands; ^3^Amsterdam UMC, University of Amsterdam, Department of Hematology, Cancer Center Amsterdam, LYMMCARE (Lymphoma and Myeloma Center Amsterdam), Amsterdam, The Netherlands; ^4^Department of Internal Medical, Division of Hematology, Maastricht University Medical Center, Maastricht, The Netherlands; ^5^Department of Hematology, Radboud University Medical Center, Nijmegen, The Netherlands; ^6^Department of Hematology, Erasmus MC Cancer Institute, Rotterdam, The Netherlands; ^7^Amsterdam UMC, Vrije Universiteit Amsterdam, Department of Hematology, Cancer Center Amsterdam, Amsterdam, The Netherlands; ^8^Department of Public Health, Erasmus University Medical Center, Rotterdam, The Netherlands

**Supplemental Tables**

**Supplemental Table 1.** Prior treatment for patients with a prior malignancy.

|  | **Number of patients** | **% of total** |
| --- | --- | --- |
| **No prior treatment** | 29 | 6% |
| **Systemic therapy without radiotherapy** | 71 | 15% |
| **Radiotherapy without systemic therapy** | 91 | 19% |
| **Systemic therapy with radiotherapy** | 69 | 14% |
| **Other therapy without systemic- and/or radiotherapy** | 223 | 46% |

**Supplemental Table 2**. Competing risk regression models for the association between a history of malignancies and development of subtypes of a second primary malignancy after a follicular lymphoma diagnosis.

|  | **Univariable** | | |  | **Multivariable^a^** | | |
| --- | --- | --- | --- | --- | --- | --- | --- |
|  | **SHR** | **95% CI** | ***P*-value^b^** |  | **SHR** | **95% CI** | ***P*-value^b^** |
| **Breast** | 1.33 | 0.62-2.86 | 0.472 |  | 1.32 | 0.60-2.91 | 0.489 |
| **Female reproductive** | 0.42 | 0.06-3.06 | 0.392 |  | 0.41 | 0.05-3.20 | 0.397 |
| **Gastrointestinal** | 1.23 | 0.72-2.12 | 0.449 |  | 1.05 | 0.60-1.84 | 0.864 |
| **Hematological** | 0.60 | 0.22-1.62 | 0.311 |  | 0.72 | 0.26-1.98 | 0.526 |
| **Kidney and urinary tract** | 0.31 | 0.04-2.25 | 0.247 |  | 0.27 | 0.04-2.02 | 0.202 |
| **Male reproductive** | 1.22 | 0.50-3.01 | 0.665 |  | 0.87 | 0.34-2.24 | 0.777 |
| **Melanoma of the skin** | 2.28 | 0.90-5.80 | 0.082 |  | 2.42 | 0.96-6.14 | 0.062 |
| **Respiratory tract** | 1.91 | 1.16-3.15 | **0.011** |  | 1.83 | 1.10-3.05 | **0.021** |
| **Squamous cell of the skin** | 2.02 | 1.31-3.12 | **0.001** |  | 1.58 | 1.01-2.45 | **0.045** |
| ^a^Multivariable models were adjusted for the following baseline characteristics: sex, age and FL stage at diagnosis, and year of FL diagnosis. | | | | | | | |
| ^b^*P*-values were compared to the reference category.  ^c^Analyses for subtypes of second primary malignancies that were rare (<5%) were omitted in this table.  Statistically significant p-values (*P*<0.05) are presented in bold. | | | | | | | |

**Supplemental Table 3.** Types of prior and subsequent malignancies among patients with follicular lymphoma.

|  | **First PMD** | **% of all PMDs** | **First SPM** | **% of all SPMs** | |
| --- | --- | --- | --- | --- | --- |
| **Bone and soft tissue** | 3 | 1% | 7 | 1% | |
| **Breast** | 123 | 26% | 97 | 9% | |
| **Endocrine** | 3 | 1% | 4 | 0% | |
| **Female reproductive** | 31 | 11% | 36 | 7% | |
| **Gastrointestinal** | 79 | 16% | 210 | 19% | |
| **Head and neck** | 13 | 3% | 28 | 3% | |
| **Hematological** | 39 | 8% | 120**^a^** | 11% | |
| **Kidney and urinary tract** | 27 | 6% | 56 | 5% | |
| **Male reproductive** | 68 | 33% | 86 | 14% | |
| **Melanoma skin** | 30 | 6% | 43 | 4% | |
| **Nervous system** | 2 | 0% | 6 | 1% | |
| **Respiratory tract** | 10 | 2% | 169 | 15% | |
| **Squamous cell skin** | 48 | 10% | 216 | 20% | |
| **Other malignancies** | 7 | 1% | 28 | 3% | |
| Abbreviations: PMD, prior malignancy diagnosis; SPM, second primary malignancy.  ^a^Follicular lymphoma patients who were diagnosed with a hematological SPM (n = 120), were diagnosed with the following entities of SPMs (n = 120): acute myeloid leukemia (n = 36), Hodgkin lymphoma (n = 19), myelodysplastic syndrome (n = 18), myeloproliferative disorder (n = 11), chronic lymphocytic leukemia or small lymphocytic lymphoma (n = 9), indolent non-Hodgkin lymphoma (n = 8), plasma cell neoplasm (n = 8), lymphoblastic leukemia/lymphoma (n = 5), mature T- and NK-cell neoplasm (n = 4), cutaneous lymphoma (n = 1), unspecified and biphenotypic acute leukemia (n = 1). | | | | |  |
